# Influenza vaccination among healthcare workers at a tertiary care hospital in Saudi Arabia: Facing challenges

**DOI:** 10.4103/1817-1737.62480

**Published:** 2010

**Authors:** Badriah M. Al-Otaibi, Aiman El-Saed, Hanan H. Balkhy

**Affiliations:** 1 *Department of Infection Prevention and Control, King Abdulaziz Medical City, Saudi Arabia*; 2 *Gulf Cooperation Council (GCC) States and WHO Collaborating Center for Infection Prevention and Control, Saudi Arabia*; 3 *King Abdullah International Medical Research Center, Riyadh, Saudi Arabia E-mail: balkhyh@hotmail.com*

Sir,

The World Health Organization (WHO) estimated that influenza virus causes worldwide severe illness in three to five million people annually, with up to half-million deaths.[1] Healthcare workers (HCWs) are at a great risk of developing influenza illness because of exposure to sick patients or other HCWs. Influenza vaccination remains the most effective measure for controlling and preventing influenza outbreak in a healthcare setting.[2] Although international guidelines require annual vaccination of HCWs against influenza,[3] influenza vaccine among HCWs remains considerably low.[4,5]

In this letter, we will review our HCWs influenza vaccination experience at King Abdulaziz Medical City (KAMC), National Guard Health Affairs, a tertiary care facility (Riyadh, Saudi Arabia) run by more than 6000 physicians, nurses and paramedical personnel. Since 2003, we have been organizing and conducting an annual seasonal influenza vaccination campaign during the months of October to February to promote influenza vaccination among HCWs. Each year the campaign would be preceded by hospital-wide advertisements using intranet, brochures, pamphlets and posters. Promotional material provided information on influenza as a disease, cough etiquette, vaccine importance and venues for free of charge vaccination.

During the first four campaigns, an outreach program to HCWs at high-risk areas was scheduled; this included: intensive care units, emergency rooms, hemodialysis unit and organ transplant units. Healthcare workers from the mentioned areas who missed these dates, as well as HCWs from any other department, were able to present to the employee health clinic. Despite using multiple overlapping announcements, vaccine coverage in the first four campaigns ranged between 21 and 29% [Figure 1].

**Figure 1:**
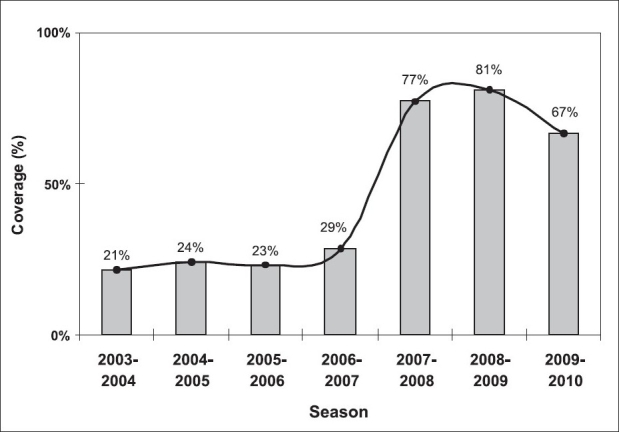
Trend of influenza vaccine coverage among HCWs at KAMC (2003–2010)

For the past three seasons, however, a new methodology was adopted. The aim was to engage the nursing department, being the largest group of clinical HCWs in the hospital, to partner in conducting the vaccination campaign. During these past three seasons, the infection control public health nurses would allow the nursing department to assign a champion to be responsible for immunizing all HCWs in his/her area. This method alone allowed for more than a two-fold increase in vaccination coverage [Figure 1].

A second major methodological change was adapted in response to the recent H1N1 influenza pandemic (April 2009). Pre-campaign advertisements and announcements stressed the mandatory rather than voluntarily nature of seasonal (2009–2010) influenza vaccine. However, HCW anxiety during the recent pandemic probably masked any benefit of this methodological change and instead we experienced a 17% drop in coverage compared to previous season. Interestingly the drop was observed only in physicians and paramedical personnel but not nurses. Analysis of the signed waiver for those who refused to get their vaccine showed that the majority had bad vaccine experience including previous reaction to influenza vaccine (55%) and allergy to eggs (13%). Moreover, 2% refused to get the vaccine because they had H1N1 during the recent pandemic.

Analysis of vaccine coverage by job category during the last three seasons [Figure 2] showed that nurses had the highest coverage rate (80%, *p* < 0.001) followed by physicians (74%) and paramedical personnel (67%). This is in contrast with some reports that showed that physicians have the highest coverage rate.[4,6] We believe that engaging nurses from all departments may have contributed to this high influenza vaccine coverage, especially among nurses.

**Figure 2:**
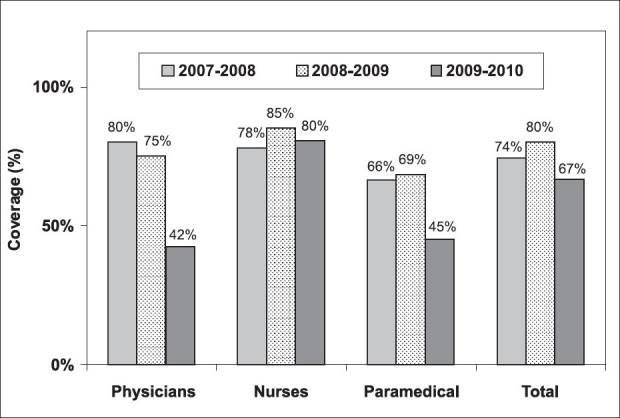
Influenza vaccine coverage among HCWs by job category at KAMC (2007–2010)

## References

[1] The World Health Organization (WHO) (2009). Seasonal Influenza, Fact Sheet N 211.

[2] Blank PR, Szucs TD (2009). Increasing influenza vaccination coverage in recommended population groups in Europe. Expert Rev Vaccines.

[3] CDC Influenza Vaccination of Health-Care Personnel. Recommendations of the Healthcare Infection Control Practices Advisory Committee (HICPAC) and the Advisory Committee on Immunization Practices (ACIP). Morbidity and Mortality Weekly Report (MMWR) Feb 24, 2006/ 55(RR02);1-16.

[4] Ballestas T, McEvoy SP, Doyle J (2009). SMAHS Healthcare Worker Influenza Vaccination Working Party. Co-ordinated approach to healthcare worker influenza vaccination in an area health service. J Hosp Infect.

[5] Maltezou HC, Maragos A, Halharapi T, Karagiannis I, Karageorgou K, Remoudaki H (2007). Factors influencing influenza vaccination rates among healthcare workers in Greek hospitals. J Hosp Infect.

[6] Abramson ZH, Levi O (2008). Influenza vaccination among primary healthcare workers. Vaccine.

